# Microhabitat choice in island lizards enhances camouflage against avian predators

**DOI:** 10.1038/srep19815

**Published:** 2016-01-25

**Authors:** Kate L. A. Marshall, Kate E. Philpot, Martin Stevens

**Affiliations:** 1 Department of Zoology, University of Cambridge, CB2 3EJ, UK; 2 Centre for Ecology and Conservation, College of Life and Environmental Sciences, University of Exeter, Penryn Campus, Penryn, Cornwall, TR10 9FE, UK

## Abstract

Camouflage can often be enhanced by genetic adaptation to different local environments. However, it is less clear how individual behaviour improves camouflage effectiveness. We investigated whether individual Aegean wall lizards (*Podarcis erhardii*) inhabiting different islands rest on backgrounds that improve camouflage against avian predators. In free-ranging lizards, we found that dorsal regions were better matched against chosen backgrounds than against other backgrounds on the same island. This suggests that *P. erhardii* make background choices that heighten individual-specific concealment. In achromatic camouflage, this effect was more evident in females and was less distinct in an island population with lower predation risk. This suggests that behavioural enhancement of camouflage may be more important in females than in sexually competing males and related to predation risk. However, in an arena experiment, lizards did not choose the background that improved camouflage, most likely due to the artificial conditions. Overall, our results provide evidence that behavioural preferences for substrates can enhance individual camouflage of lizards in natural microhabitats, and that such adaptations may be sexually dimorphic and dependent on local environments. This research emphasizes the importance of considering links between ecology, behaviour, and appearance in studies of intraspecific colour variation and local adaptation.

Populations of the same species often exhibit colour variation among distinct local environments. A recent body of work, in particular on lizards and mice, has shown that genetic adaptation to local environments can cause intraspecific variation in background-matching camouflage among different populations^1^,^2^,^3^,^4^. In addition to these fixed adaptations, behaviour may also optimize camouflage across varying local environments. As camouflage depends on the visual background against which it is viewed, both classical and more recent evidence suggests that various species, and discrete colour morphs within species, prefer habitats and backgrounds that heighten matching for camouflage, including in insects, reptiles and fish (e.g.^5^,^6^,^7^,^8^,^9^,^10^,^11^,^12^,^13^). For example, bark-resting moths prefer habitats that have backgrounds with similar colour and luminance to that of their own appearance^13^,^14^ and can re-position their resting body orientations to enhance camouflage^7^,^8^.

However, studies have rarely tested whether individuals within a species showing continuous variation in appearance actively choose specific backgrounds, and whether these choices actually heighten the effectiveness of their own camouflage against predators. To our knowledge, only one recent study has conducted a comprehensive test of this question^15^. This found that individual ground-nesting Japanese quail (*Coturnix japonica*) given a choice between four differently coloured (artificial) substrates typically chose the background that provided optimal camouflage for their eggs. This suggests that, because quail eggs vary substantially in appearance among mothers, individuals are able to choose substrates that optimise individual camouflage. Yet, although this study used strictly controlled backgrounds and image analysis methods to objectively quantify egg and substrate appearance, measurements of egg camouflage were based on human metrics of appearance in an artificial setting. Thus, this study did not directly reveal whether behaviour optimizes camouflage to actual predators in the wild. This is important because predators often have different visual systems to that of humans and thus will perceive behaviourally enhanced camouflage differently (e.g.^9^) and because most natural substrates are highly variable and complex. Therefore, here we aimed to test whether wild individuals choose natural backgrounds to make themselves less detectable to potential predators in order to clarify whether background choices adaptively heighten camouflage.

We addressed this in island populations of Aegean wall lizards (*Podarcis erhardii*) in Greece, which show continuous variation in coloration both at the population level (among-island) and at the individual level (within-island). The evolutionary and ecological causes of the among-island colour variation in *P. erhardii* have already been investigated in three recent studies^16^,^17^,^18^. Key results from these studies showed that avian predators perceive island populations of *P. erhardii* to be better camouflaged against their native island rock backgrounds than against the rock backgrounds of different islands, indicating that among-island (dorsal) colour variation has been caused by local adaptation for camouflage, presumably via natural selection^16^. Additionally, in most populations, females are generally camouflaged while sexually competing males tend to have more conspicuous coloration on signalling body regions, indicating sexual dimorphism^17^. Subsequent survival experiments with artificial models showed that, in one island population, conspicuous males suffered increased attacks by predatory birds compared to relatively camouflaged females, indicating a selective advantage of crypsis at the population level^18^.

Therefore, the current research differs from other recent studies of *P. erhardii* because, instead of focusing on colour adaptation at the population level (among-island variation; see^16^,^17^,^18^), here we investigated colour variation and behaviour at the individual level (within-island variation). Specifically, we examined whether behavioural background choices made by differently coloured individuals enhanced their own degree of camouflage in specific microhabitats, which is currently unknown.

In general, a substantial amount of research on adaptive behavioural plasticity in lizards has shown links between (human-assessed) coloration and camouflage and adjustments of anti-predator behaviours, such as escape decisions (e.g.^19^,^20^,^21^,^22^,^23^). Moreover, some studies have suggested that individual lizards have preferences for backgrounds that better match their dorsal coloration. For example, laboratory experiments testing lizards’ preferences for different artificial backgrounds have shown that individuals varying in colour prefer substrates that appear to better match their coloration (to human vision), such as in geckos and iguanids^24^,^25^,^26^. However, little is known about whether individual lizards in the wild choose natural backgrounds to enhance their own degree of camouflage, as perceived by likely predators.

Although camouflage appears to change seasonally in *P. erhardii* (K. Marshall, unpublished data), they are not capable of rapid colour changes to alter camouflage against particular substrates, as in other lizards such as chameleons and geckos (e.g.^27^,^28^). Instead, background choices may allow individual *P. erhardii* to flexibly alter camouflage according to the local environment and ecological context (e.g. perceived threat from predators), particularly as other lizards can adjust their anti-predator behaviours in relation to immediate predation risk (e.g.^29^,^30^). *P. erhardii* are typically seen basking on rock substrates in open environments, as in many other rock-dwelling lizards^31^,^32^, where they are probably highly visible to many resident avian predators, such as raptors (e.g. buzzards, *Buteo buteo* and falcons, *Falco eleonorae, Falco tinnunculus*) and corvids (e.g. hooded crows, *Corvus cornix*)^33^. This indicates strong selection for behaviours that optimize camouflage on exposed dorsal regions, and particularly on the posterior dorsal region in *P. erhardii*, because the anterior region appears to have a conflicting function in sexual signalling^16^,^17^.

The different islands that these lizards inhabit have distinctive ecological and biogeographic characteristics. For example, some islands have lower numbers of avian predator species than others^33^, and some are land-bridge islands that have been separated from the mainland for ≈12,000y while others have been formed through a prolonged (>200,000y) history of volcanic eruptions (see^16^). Accordingly, the inhabitant lizard populations show varying degrees of camouflage among these different environments, with some populations showing better local camouflage than others^16^. Therefore, studying this island system can reveal whether individuals of different populations choose resting sites that improve camouflage and thus whether its occurrence is dependent on the general ecological characteristics of each island. For example, past studies have shown that anti-predator (escape response and tail autotomy) behaviours are reduced in relaxed predation environments in insular lizard populations, including in *P. erhardii*^34^,^35^, suggesting that lower degrees of predation pressure may reduce the need for background choices to improve camouflage.

Here, we examined the role of substrate choice in improving background-matching camouflage against avian predators. In free-ranging individual lizards, we measured differences in camouflage between their chosen backgrounds and other backgrounds on the same island. Moreover, we performed an arena experiment in which we tested whether lizards actively chose to rest on one background over another to heighten their own camouflage (see Fig. 1). In free-ranging lizards, we predicted better dorsal background matching against chosen backgrounds than against other, non-chosen backgrounds. We also predicted that behavioural enhancement of camouflage would be more evident in females than in sexually competing males. Furthermore, we predicted that the degree of behaviourally enhanced camouflage would vary among island populations differing in predation risk and habitats. In the arena experiment, we predicted that lizards would more frequently choose to rest on the background that heightened dorsal camouflage against avian predators, and that stronger background preferences would be linked to heightened camouflage.

## Methods

### Study sites and species

The Aegean wall lizard (*Podarcis erhardii*) is a diurnal, small lacertid distributed across most of the South Balkans and widespread throughout many Aegean islands where it is abundant in all ecosystems^36^. We conducted field research during the main activity season (April–July^37^) in 2012 and in 2013 on four Aegean islands: Folegandros (36°37′ N, 24°54′ E), Santorini (36°25′ N, 25°26′ E), Syros (37°27′ N, 24°54′ E), and Skopelos (39°7′ N, 23°43′ E). Arena experiments were carried out on Folegandros, Skopelos and Syros in 2013.

### Ethics statement

All methods and experimental protocols as described below were in accordance with the policies and requirements of the University of Cambridge ethics committee, and with guidelines and regulations specified by the Association for the Study of Animal Behaviour. In particular, in the arena experiments, we used capture and handling techniques that did not harm the lizards, which all showed normal behaviour after release. This research did not require any authorization under the UK Animals (Scientific Procedures) Act 1986 because fieldwork was conducted abroad and the study species, *Podarcis erhardii*, is not a protected species. We conducted all research with permission from the Greek Ministry of Environment (permit number: 166648/356) and all land used for fieldwork was publicly accessible. All experimental protocols were approved by the ethics committee of the School of Biological Sciences, University of Cambridge.

### Photography of free-ranging lizards and their backgrounds

As in past work, we used digital imaging to sample lizards and background coloration^17^,^38^,^39^. We took *in situ* images of stationary lizards and their natural corresponding backgrounds with a Fujifilm IS Pro ultraviolet (UV)-sensitive digital camera with a quartz CoastalOpt UV lens (Coastal Optical Systems), fitted with a UV and infrared (IR) blocking filter for photographs in the human-visible spectrum (Baader UV/IR Cut filter; transmitting between 400 and 700 nm), and with a UV pass filter (Baader U filter; transmitting between 300 and 400 nm) for UV images. After the photographed lizard had fled, we took human-visible and UV images of a Spectralon^TM^ grey reflectance standard (Labsphere, Congleton, UK), which reflects light equally at 40 per cent between 300 and 750 nm, under the same light conditions as the lizard to standardize photographs for ambient light conditions (see^40^,^41^).

We recorded the location of the photographed free-ranging lizards using a Garmin eTrex® GPS device (Schauffhausen, Switzerland) and used a field guide to estimate their sex and lifestage^36^. In other studies, we have tested the reliability of our estimations of sex and lifestage in free-ranging lizards (K. Marshall, 2013, unpublished data). To do this, we first photographed free-ranging lizards and estimated their sex and lifestage from field observations and photographs. We then captured the same individual to determine actual sex and lifestage (by identifying femoral pores on hindlegs and hemipenal bulges in adult males) to check the accuracy of our initial estimate. We found that the estimated and actual scores were the same in 99% of cases. Therefore, this suggests that our estimations of sex and lifestage in free-ranging lizards are highly reliable (K. Marshall, 2013, unpublished). We avoided pseudoreplication by never repeating photography of a lizard of the same sex within the same home range (i.e., within 10 m) (see^17^,^42^).

### Image analysis and visual modelling

We followed methods previously described in past work^17^. Human-visible and UV images of lizards and backgrounds were linearized with respect to light intensity and transformed to reflectance (RGB-equalized) (see^38^,^39^). Any images that were overexposed and/or could not be RGB-equalized were discarded from the analysis.

We used a mapping process based on the spectral sensitivity of our camera’s sensors to convert the images to correspond to avian predicted photon catch cone values, which is highly accurate compared to photon catch estimates derived from reflectance spectrometry (see^38^,^39^,^43^). We converted the aligned images from camera colour space to the relative photon catches of an avian predator’s longwave (LW), mediumwave (MW), shortwave (SW) and UV sensitive cone photoreceptors, using the spectral sensitivity of the peafowl (*Pavo cristatus*^44^). We also transformed the images to correspond to the relative photon catches of a bird’s double cones, which encode luminance (achromatic) information, again using the spectral sensitivity of *P. cristatus*^45^. The peafowl visual system is often used as a model of the violet-sensitive (VS) class of avian colour vision, which is typical of predatory raptors and corvids^46^ that are major visual predators of *Podarcis* lizards and other small lacertids in Europe^33^,^47^. We acknowledge that ultraviolet-sensitive (UVS) predators may also prey on *P. erhardii*, such as gulls and some Turdidae species present in Greece^33^,^48^,^49^. However, these birds were rarely seen in our study sites whereas VS raptors and corvids were typically seen on a daily basis and therefore were treated as the major predator type (personal observations). Calibrations were performed in MATLAB v. R2011b (The MathWorks, Inc., MA, USA) using custom-written programs and were restricted to the 30–700 nm range, which encompasses most of the visual spectrum of diurnal birds^50^.

LW, MW, SW and UV photon catches of lizards and their backgrounds were extracted from the calibrated images in ImageJ using the selection tool. Each selection generated an average photon catch value for a selected patch. Background selections were limited to rocks, avoiding areas of lichen and moss, and were made in areas adjacent to but not overlapping the lizard. We aimed to select a background area that most represented the colour of the rock on which the lizard had chosen to rest. We identified the largest (predominant) colour patch that was adjacent to the lizard and made our selection from that area. However, occasionally one predominant colour patch could not be distinguished. In these cases, we made selections of up to three different colour patches on the rock, and averaged the photon catches across these regions to determine a representative background colour.

Lizard selections were made from two dorsal body regions (posterior lower and anterior upper backs). Selections included any dorsal patterning to measure the appearance of the dorsal surface visible to birds hunting from a distance. Specifically, lower back selections were taken at the base of the tail and upper back selections were taken at the base of the head (see^17^).

### Background matching

We analysed how effectively adult lizards matched the backgrounds we photographed them resting on (hereafter ‘own’ chosen backgrounds) versus the backgrounds of other lizards on the same island (hereafter ‘other’ backgrounds). We quantified colour contrasts between photon catches of lizards and photon catches of backgrounds according to the log form of a widely used receptor noise model^51^, which predicts visual discrimination abilities in observers. We also quantified luminance contrasts using a version of the model based on achromatic differences using peafowl double cones^52^. To account for receptor noise, we used a Weber fraction value of 0.05 for the most frequent cone type based on data in other vertebrates^51^,^53^. We used relative proportions of cone types in the peafowl retina to calculate avian predator-perceived chromatic contrast (i.e., LW = 0.92, MW = 1.00, SW = 0.81, UV = 0.54)^44^.

The degree of chromatic and luminance contrast generated from these models is expressed in “just-noticeable-differences” (JND). JND values between 1.00 and 3.00 indicate difficult discrimination except under optimal light conditions, while values increasing above 3.00 indicate increasingly improved discrimination^52^. For each image, we calculated how well avian predators could distinguish a lizard’s back against both its own and other backgrounds (JND). We obtained two JND values for each body region for each individual lizard. Specifically, one JND value represented individual lizard contrast against its own background and the other represented its (average) contrast against every other lizard’s background on the same island. Overall, this was acquired in 263 free-ranging adult lizards from the four focal island populations (Folegandros = 100; Syros = 49; Santorini = 58; and Skopelos = 56 [149 males, 114 females]).

### Arena experiment

#### Experimental set-up

**Figure 1:**
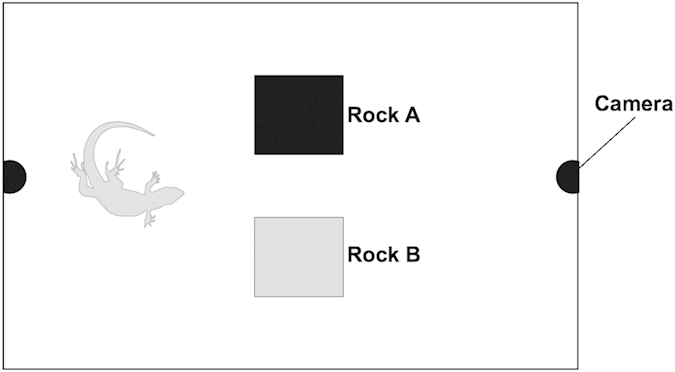
Representative drawing of the arena experiment set-up. Two differently coloured rocks (rock A and rock B) were placed side-by-side in the centre of the enclosure. Video cameras recorded background choices made by each lizard while experimenters were out of sight. In subsequent video analysis, if the lizard showed better camouflage against one rock than the other rock as perceived by avian predators (JND) then it was expected to spend a greater proportion of time on that rock. This would indicate a background choice to enhance individual camouflage.

We used an acrylic open-topped enclosure (64 × 46 × 42 cm) covered with fine mesh netting to allow in air and natural light. The enclosure was always situated close to the original capture location in undisturbed areas to limit stress during capture. We placed a pair of experimental backgrounds consisting of two differently coloured rocks in the centre side-by-side (Fig. 1). We used rocks from local environments as natural substrates typically used by lizards (e.g.^31^) and to keep background type consistent with that of our study on free-ranging lizards. The same pair of (local) rocks was used in all trials on each island so that each individual of each population had the same background choice across trials. This allowed us to compare the relative camouflage of each lizard against the two backgrounds, and thus how this affected background choices and strength of background preferences between different individuals (see video and statistical analyses).

We used rock pairs with approximately equal surface area (cm^2^). We verified each individual lizard’s degree of camouflage against both backgrounds once the experiment had finished. To do this, we obtained avian predator photon catches from calibrated images of experimental backgrounds and calculated the chromatic and luminance contrast (JND) between the backgrounds and each individual lizard using the receptor-noise model as above^51^.

To remove any conspecific and human scents, we handled experimental backgrounds with gloves and cleaned them and the enclosure before use in each trial. We placed rocks in the same position, with the photographed surface facing upwards, and under the same amount of natural light to standardise basking conditions. Before starting the trial, we measured background temperatures using a Pellor Victor 303B infrared laser thermometer gun (Shenzhen City, China) with an accuracy of ±1.5 °C and resolution of 0.1 °C.

#### Experimental procedure

For each trial, we captured a different lizard using a noose. After capture we immediately obtained human-visible and UV photographs of the dorsal body region using the described photography procedure. The lizard was placed onto the floor of the enclosure, always in the same place, to start the trial. We immediately moved out of sight and used two mini DK-650 DVR video cameras (Shenzhen DKSECU Technology Co. Ltd, Guangdong, China) to record behaviours (see Fig. 1). We stopped the trial after 20 minutes because pilot studies had shown this was the minimum time necessary for the lizards to acclimatise and begin to use the experimental substrates.

Before release, we marked lizards on the hind leg with water-based acrylic paint (Montana, Heidelberg, Germany). After marking we released the lizard at its original capture location. Observations during fieldwork showed that the paint marks were visible in free-ranging lizards (from a maximum distance of ≈3 m) and in other mark-recapture studies they were found to last for at least three weeks (K. Marshall, 2012–2013, unpublished data). As capture and release periods on each island did not exceed three weeks in the current study, we assumed, based on our previous observations, that the marks would persist during this time so as to reliably indicate whether individuals had been previously captured. Additionally, once a successful capture and release had been made in one location, we then moved to another non-adjacent site to avoid capturing the same individual, and never repeated capture attempts in the same location. Indeed, during fieldwork we did not observe paint markings on captured or on free-ranging lizards, which suggests that we avoided encountering previously caught individuals and prevented pseudoreplication. A total of 71 experimental trials was conducted overall. However, in video playback, we found that some lizards hid under the rocks throughout the trial. This meant that we observed lizards using the experimental backgrounds in just 58 trials and therefore only these trials were included in the analysis (Folegandros = 28, Skopelos = 21 and Syros = 9 [12 females and 46 males]).

#### Video analysis

In video analysis, we timed periods when the lizard’s head and body was observed against a substrate, and stopped timing when this did not occur. For each trial, we calculated how much time the lizard spent on each background as a proportion of the total time spent resting on both. We identified which background the lizard had spent the majority of time on (i.e., >50–100%) to indicate a chosen substrate. However, we recognized that this was an arbitrary point at which to assume a choice had occurred. Therefore, in subsequent analyses we increased the point at which we assumed a background choice had been made (i.e., >50%, ≥60%, ≥70%, ≥80% and ≥90% of the proportion of the total time). Moreover, we also considered how these increasing proportions of time spent on the background (i.e., preference ‘strength’) were related to background choices.

Furthermore, in each trial, we determined whether a background choice could potentially benefit camouflage by verifying whether the lizard was indistinguishable against one background to avian predators under natural light conditions (i.e., ≤3.00 JND)^52^. These trials were scored as having a “benefit ” to camouflage, whereas trials where lizards were >3.00 JND against both backgrounds were scored as having “no benefit”. There was just one trial in which the lizard was indistinguishable against both backgrounds (i.e., ≤3.00 JND). We scored this trial as “no benefit” because we assumed that there would be no (relative) camouflage benefit in choosing one background over the other.

In the “benefit” group, we scored trials based on whether the lizard had achieved camouflage (≤3.00 JND) or no camouflage (>3.00 JND) through its background choice. This provided frequencies of trials categorized into two groups based on the choice outcome (1 = camouflaged and 2 = not camouflaged). In the “no benefit” group, we considered whether the proportion of time spent on the chosen background (i.e., preference strength) was lower than in the “benefit” group. Finally, in both “benefit” and “no benefit” groups, we assessed whether preference strength was related to the individual’s degree of contrast (JND) against their chosen background.

### Predictions and statistical analyses

#### Free-ranging lizards

Normality tests and residuals analysis showed that the colour and luminance JND data were not normally distributed. Therefore, these datasets were transformed using logarithmic and square root transformations, respectively. Raw back-transformed data are reported in figures as mean and S.E. values.

We predicted that within each island population, camouflaging dorsal regions would be perceived by avian predators as better matched against their own (chosen) backgrounds than against other lizards’ backgrounds on the same island. In addition, we predicted that behavioural enhancement of camouflage would be a) more likely in females than in males, b) more likely in lower backs than in upper backs, and c) less likely in an island population with relatively fewer resident avian predator species (Folegandros^33^).

To test these predictions, we conducted two separate mixed general linear models (GLMs). The first tested chromatic JND data (Test 1) and the second tested luminance JND data (Test 2). Both tests had four variables: island population (Folegandros, Santorini, Skopelos and Syros) and sex included as between-subjects factors, and background comparison (own versus other backgrounds), and body region (lower backs versus upper backs) were included as within-subjects factors. We report JND scores as means ± S.E. values and report the size of the effects in partial ETA^2^ (η_p_^2^), which can be interpreted as the proportion of variance in the dependent variable that is attributable to each effect. In all GLMs, we tested for factor interactions that addressed our predictions. Planned comparisons were conducted that were relevant to our predictions by re-running the GLMs with only the factors of interest included, limiting the number of comparisons to the number of “spare” degrees of freedom (n − 1) because these are more powerful than conservative post hoc comparisons^54^. Factors with non-significant effects were removed from the analysis before re-running the GLM.

#### Arena experiment

Normality tests and residuals analysis showed that the substrate temperature (°C) data had a normal distribution. However, the preference strength data (i.e., the proportion of time spent resting on the chosen background) was not normally distributed and was resistant to transformation to normality so we performed non-parametric tests on this data.

We conducted a parametric paired samples *t*-test to verify whether mean temperature differed between lizards’ chosen and not chosen backgrounds. We analysed the scored frequency data (grouped into 1 = “camouflaged” and 2 = “not camouflaged”) using binomial tests. These analysed our prediction that individuals would more frequently choose the camouflaging over the non-camouflaging background in more cases than would be expected by chance, with the test proportion set to 50% of cases.

In addition, two-tailed Mann-Whitney *U* tests assessed whether lizards that had chosen the camouflaging background showed a stronger preference for that background compared to lizards that had chosen the non-camouflaging substrate. These tests also examined whether weaker preferences occurred in the “no benefit” group compared to the “benefit” group. Finally, in both the “benefit and “no benefit” groups, we carried out two-tailed Spearman’s *rho* correlation tests to determine whether preference strength for a background was associated with a lizard’s degree of contrast against that background (JND). Specifically, we predicted that in the “benefit” group, preference strength would increase with decreasing JNDs, whereas in the “no benefit” group, we predicted that JNDs and preference strengths would be unrelated. In all statistical analyses, we rejected the null hypothesis when *P* ≤ 0.05.

## Results

### Test 1: Chromatic background matching in free-ranging lizards

**Figure 2:**
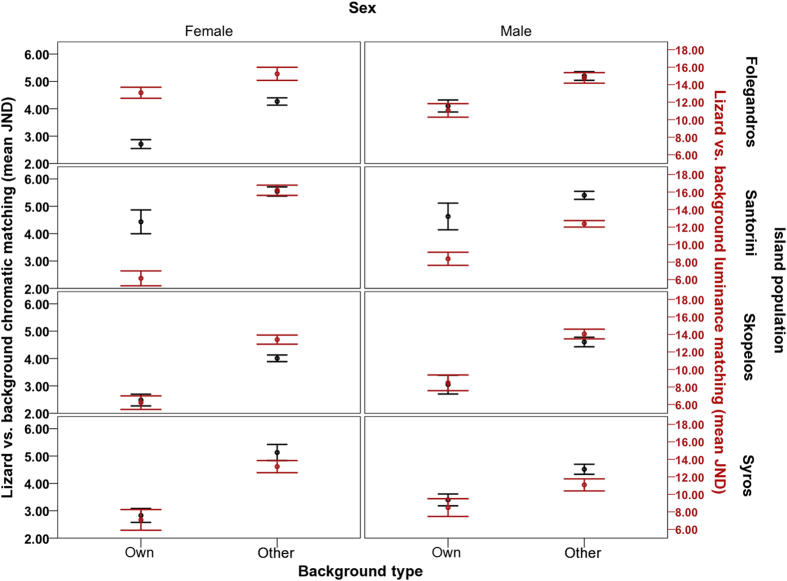
Background-matching camouflage in free-ranging lizards. This shows the degree of chromatic (left axis; black data points) and luminance (right axis; red data points) background matching (mean JND ± 1.00 S.E) of the dorsal regions of free-ranging Aegean wall lizards (*Podarcis erhardii*). Dorsal matching against ‘own’ chosen backgrounds (rock substrates the lizards were observed resting on) and against other lizards’ backgrounds (other) on the same island is shown in four island populations (Folegandros, Santorini, Skopelos and Syros) and in males and females (*N* = 263; Folegandros = 100; Syros = 49; Santorini = 58; and Skopelos = 56 [149 males, 114 females]). Generally, values ≤3.00 JND depict lizards that are indistinguishable (camouflaged) against the background to avian predators under natural lighting conditions,, while values increasing >3.00 JND depict lizards that are increasingly distinguishable.

The GLM reported a highly significant difference in chromatic background matching (JND) of *P. erhardii* backs between comparisons with chosen backgrounds and other lizards’ backgrounds on the same island, as perceived by avian predators (F_1,255_ = 166.418, *P* < 0.001; Fig. 2). This effect accounted for most of the variance in the model (η_p_^2^ = 0.395). Specifically, in all island populations and in both males and females, both dorsal regions of *P. erhardii* were significantly more camouflaged to avian predators against their own chosen backgrounds than against other lizards’ backgrounds (Fig. 2; own = 3.500 ± 0.110 vs. other = 4.805 ± 0.063). As predicted, this effect was particularly distinct in lower backs compared to upper backs (F_1,255_ = 8.271, *P* = 0.004, η_p_^2^ = 0.031; lower backs [own vs. other; 3.180 ± 0.154 vs. 4.578 ± 0.083; upper backs: 3.819 ± 0.156 vs. 5.033 ± 0.093).

### Test 2: Achromatic background matching in free-ranging lizards

As in the chromatic visual model, the GLM reported a highly significant difference in achromatic background matching of *P. erhardii* backs between comparisons with their own backgrounds and other backgrounds on the same island (F_1,255_ = 154.586, *P* < 0.001; Fig. 2). Again, this effect accounted for most of the variance in the model (η_p_^2^ = 0.377). As predicted, *P. erhardii* were significantly more camouflaged to avian predators against their own chosen backgrounds than against other backgrounds on the same island (Fig. 2; own = 9.291 ± 0.315 vs. other = 13.890 ± 0.241 JND), although this effect was dependent on island population and sex (background comparison*island population: F_3,255_ = 7.597, *P* < 0.001, η_p_^2^ = 0.082; background comparison*sex: F_1,255_ = 5.837, *P* = 0.016, η_p_^2^ = 0.022; Fig. 2). These effects were analysed in planned comparisons described below.

#### *In situ* achromatic background matching: sexual dichromatism and island differences

As predicted, the extent to which individual lizards selected backgrounds to improve achromatic camouflage differed between island populations. Specifically, achromatic camouflage against own backgrounds compared to other backgrounds was less distinct in the Folegandros population (F_1,98_ = 17.397, *P* < 0.001, η_p_^2^ = 0.151 [own = 12.030 ± 0.507 vs. other = 15.000 ± 0.480]) compared to the other island populations, particularly Skopelos and Santorini (Fig. 2; Skopelos: F_1,54_ = 68.133, *P* < 0.001, η_p_^2^ = 0.558 [own vs. other JND; 7.384 ± 0.603 vs. 13.740 ± 0.381]; Santorini: F_1,56_ = 55.093, *P* < 0.001, η_p_^2^ = 0.496 [7.454 ± 0.568 vs. 13.960 ± 0.369]; Syros: F_1,47_ = 24.600, *P* < 0.001, η_p_^2^ = 0.344 [8.060 ± 0.790 vs. 11.721 ± 0.530]).

In addition, as predicted, achromatic camouflage in both females and males was enhanced against their own backgrounds as opposed to other lizards’ backgrounds overall. However, this effect was more pronounced in females than in males, in terms of η_p_^2^ (Fig. 2; females: F_1,110_ = 95.187, *P* < 0.001, η_p_^2^ = 0.464 [own vs. other JND; 9.203 ± 0.454 vs. 14.737 ± 0.378]; males: F_1,145_ = 60.810, *P* < 0.001, η_p_^2^ = 0.295 [9.359 ± 0.435 vs. 13.242 ± 0.307]).

### Arena experiment

The *t-*test showed there was no difference in temperature between the chosen and non-chosen backgrounds overall (*t*_64_ = −0.863, *P* = 0.392; chosen = 30.598 ± 0.520 °C vs. not chosen = 30.803 ± 0.607 °C). The binomial tests showed that, in trials where lizards could potentially improve their own degree of camouflage, there was no difference in the frequency of background choices that heightened camouflage compared to those that did not (*chromatic:* lower backs: *P* = 0.265, [number of camouflage vs. no camouflage trials; 11 vs.18]; upper backs: *P* = 1.000 [8 vs. 8]; *achromatic:* lower backs: *P* = 0.629 [10 vs.7], upper backs: *P* = 0.678 [13 vs.10]). These results did not change if we increased the proportion of time spent on the background as an indicator of background choice (i.e., >50%, ≥60%, ≥70%, ≥80% and ≥90% of the proportion of the total resting time). Therefore, contrary to our predictions, lizards spent the main proportion of time on one background irrespective of whether that background enhanced camouflage.

In addition, two-tailed Mann-Whitney *U* tests showed that preference strength (i.e., median proportion of time spent on one background between >50–100% of the total resting time) unexpectedly did not differ between choices that improved camouflage and choices that did not (*chromatic*: lower backs: *U*_(27)_ = 98.000, *P* = 0.964, [camouflaged vs. not camouflaged; 72% vs. 73%]; upper backs: *U*_(14)_ = 31.000, *P* = 0.916 [72% vs. 79%]; *achromatic:* lower backs: *U*_(15)_ = 27.500, *P* = 0.463 [70% vs. 76%]; upper backs: *U*_(21)_ = 53.000, *P* = 0.456 [85% vs. 78%]). That is, lizards that chose the camouflaging background typically spent a similar proportion of time on it as other lizards that chose the non-camouflaging background.

**Figure 3:**
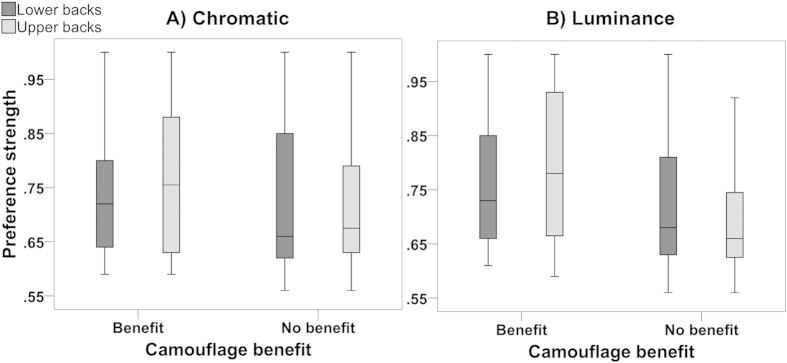
Arena experiment: Differences in preference strength between trials with “benefit” and “no benefit” to camouflage (N = 58). The largest proportion of time Aegean wall lizards (*Podarcis erhardii*) spent on one of two backgrounds (out of the total time spent on both) indicated the strength of the preference for that background. Trials provided a camouflage “benefit” when individuals were indistinguishable against one background to avian predators (i.e., ≤3.00 JND). Other trials provided “no benefit” to camouflage because lizards were distinguishable against both backgrounds to avian predators (i.e., >3.00 JND).

Further two-tailed Mann-Whitney *U* tests revealed that, in general, the median proportion of time spent on the chosen background was no different between the “benefit” and “no benefit” groups (Fig. 3; *chromatic*: lower backs: *U*_(56)_ = 372.000, *P* = 0.450 [benefit vs. no benefit; 72% vs. 66%], upper backs: *U*_(56)_ = 276.500, *P* = 0.300 [76% vs. 68%]; *achromatic:* lower backs: *U*_(56)_ = 273.000, *P* = 0.197 [73% vs. 68%]). That is, contrary to our predictions, preference strength for a certain background was the same irrespective of whether or not lizards’ background choices could heighten chromatic upper and lower back camouflage and achromatic camouflage of lower backs. The only statistically significant finding from this analysis was that, as predicted, lizards generally had stronger preferences for their chosen backgrounds in trials where a background choice could enhance achromatic camouflage of their upper backs, compared to trials in which there was no equivalent benefit to camouflage (Fig. 3; *U*_(56)_ = 233.500, *P* = 0.007 [benefit = 78% vs. no benefit = 66%).

Finally, and also contrary to our predictions, two-tailed Spearman’s *rho* correlations revealed that in the “benefit” trials, in which lizards could improve camouflage through a background choice, there was no association between the proportion of time spent on the chosen background (preference strength) and the degree of contrast between the individual lizard and that background (JND) (*chromatic:* lower backs: *r*_*s* (27)_ = −0.136, *P* = 0.483, upper backs: *r*_*s* (14)_ = 0.092, *P* = 0.734; *achromatic:* lower backs: *r*_*s* (15)_ = 0.203, *P* = 0.434, upper backs: *r*_*s* (21)_ = −0.103, *P* = 0.640; data not figured). However, as predicted, in the trials in which neither background provided camouflage, there was again no significant association between preference strength and degree of contrast (JND) against the chosen background (*chromatic:* lower backs: *r*_*s* (27)_ = 0.003, *P* = 0.987, upper backs: *r*_*s* (40)_ = −0.019, *P* = 0.903; *achromatic:* lower backs: *r*_*s* (39)_ = 0.105, *P* = 0.515, upper backs: *r*_*s* (33)_ = 0.022, *P* = 0.899; data not figured).

## Discussion

In this study, we investigated whether individual Aegean wall lizards (*Podarcis erhardii*) inhabiting different islands behaviourally enhance their own camouflage against avian predators. Our results strongly suggest that free-ranging lizards chose resting sites in their local microhabitat that improved dorsal camouflage, relative to other lizards’ backgrounds on the same island. However, in the arena experiment, lizards did not appear to actively choose one background over another in relation to their degree of camouflage.

As predicted, within the same island, free-ranging individuals of all four focal island populations exhibited enhanced dorsal matching against their own (chosen) rock backgrounds than against other lizards’ rock backgrounds, as perceived by avian predators (in terms of both chromatic and achromatic contrast; Fig. 2). Moreover, dorsal chromatic contrast was often <3.00 JND on average in females, indicating that they were probably indistinguishable against their chosen backgrounds to avian predators under natural light conditions^52^ (Fig. 2). These findings strongly suggest that lizards rest on backgrounds that heighten their own dorsal camouflage to reduce detection by avian predators. Although the exact mechanism underlying our findings requires further work, to our knowledge this is the first evidence of its kind in wild lizards. Furthermore, the only past study that has comprehensively tested individual background choices to improve crypsis used artificial backgrounds in a laboratory experiment and was based on human detection data^15^. Therefore, our findings appear to be the first demonstration of this occurring in wild populations as viewed by a typical predator visual system.

Our results are also substantiated by other laboratory experiments showing how (human) assessed lizard coloration and camouflage can be related to background preferences^24^,^25^,^26^ and adjustments of anti-predator behaviours and habitat preferences^21^,^23^,^55^. Moreover, evidence from other taxa suggests that certain other behaviours can enhance individual camouflage, such as re-positioning of body orientation in bark-resting moths^7^,^8^ to facilitate reduced detection by actual predators^9^. Thus, past evidence supports the likelihood that *P. erhardii* can modify their behaviour to improve their own camouflage.

This ability is likely to be favoured by selection because, as in other rock-dwelling lizards, *P. erhardii* rest in open areas on rocks where they are potentially visible and at risk of attack by hunting birds, especially if they are conspicuous^18^. Exposed dorsal regions are highly visible to aerially hunting birds and thus require increased camouflage compared to other less exposed regions in local environments^16^,^17^,^56^. Without the ability to rapidly change colour, choosing to rest on certain backgrounds may allow *P. erhardii* to flexibly heighten dorsal camouflage according to the immediate ecological context (e.g. perceived threat from predators), similarly to other risk-dependent anti-predatory decisions in other lizards^29^,^30^.

Our results also showed that lower (posterior) backs of both sexes, and in females more generally, were better camouflaged against chosen backgrounds relative to other backgrounds compared to that found in upper (anterior) backs and males (Fig. 2). Previous work in *P. erhardii* and other lizards has shown that camouflage is more important in females (e.g.^17^,^56^) and that lower backs of *P. erhardii* function in camouflage while upper backs are relatively conspicuous in males and possibly function as sexual signals^17^,^18^. These results suggest that, as predicted, females and lower backs are better adapted to optimize camouflage in local microhabitats, and that behavioural enhancement of upper back camouflage in males may be constrained by their simultaneous function in sexual signalling (although only in terms of chromatic contrast; Fig. 2).

In addition, relatively heightened achromatic camouflage against chosen backgrounds was reduced in Folegandros lizards compared to that of other focal populations. Lizards on this island potentially experience lower predation risk due to the fewer number of resident avian predators^16^,^33^, which may have weakened selection for behavioural enhancement of achromatic camouflage in this population. Indeed, past work has shown that *P. erhardii* and other island lizards show reduced anti-predator behaviours in relaxed predation environments^34^,^35^. Thus, local ecological factors may play an important role in the occurrence of behavioural background choices that improve camouflage.

However, the arena experiment did not support the results in the free-ranging lizards. Captive lizards did not more frequently choose the background that would make them less detectable to an avian predator under natural light conditions (i.e., ≤3.00 JND)^52^. Moreover, the strength of lizards’ background preferences (i.e., the proportion of time they spent resting on one of the two backgrounds) was unrelated to whether that background provided better camouflage or not. Thus, in these artificial captive situations at least, lizards did not actively choose a background to enhance their own camouflage.

These unexpected findings are probably due to the artificial conditions of the experiment, and because trials lasted for only the minimum time it took the lizards to acclimatise. Furthermore, the captive population used in the experiment may have exhibited unnaturally high levels of risk-taking behaviours, given that captured lizards are more likely to be bolder^57^,^58^,^59^ and that the majority of trials (79%) used male individuals, which have conflicting needs to heighten both camouflage and conspicuous sexual signals for intraspecific communication^17^. Therefore, further work addressing these issues may still find corroborating experimental evidence.

In principle, the unexpected experimental results might indicate that our sample of free-ranging lizards was biased towards only the surviving and thus better camouflaged individuals and that our findings could be due to higher predation rates on mismatched individuals^18^,^60^. However, this is highly unlikely because lizards quickly move around over multiple substrates, and so can actively seek refuges or select a better matching background under predation risk. This further suggests that, without unnaturally rapid and extremely intense predation rates, the free-ranging lizards we photographed in our study were more camouflaged due to behavioural substrate choices rather than through different survival rates.

Our key findings suggest that Aegean wall lizards (*Podarcis erhardii*) choose backgrounds that enhance matching for camouflage against avian predators in their natural microhabitats. Our results provide the first known demonstration of this occurring in wild populations as viewed by actual predators, and in lizards specifically. Further work is needed to understand the exact mechanism underlying our findings. For instance, genetic control combined with visual input may underlie adaptive background choices, and animals may learn to discriminate between camouflaging and non-camouflaging backgrounds. In addition, future experiments should investigate whether substrate choices that improve camouflage actually benefit survival. Finally, it would be valuable to examine whether background choices can be flexibly adjusted for multiple functions in camouflage, thermoregulation and sexual signalling. Overall, our results indicate that individual behaviours have an important role in enhancing camouflage across different environments. In our island system, we further reveal that these behavioural adaptations may be habitat-dependent, possibly allowing animals to flexibly adjust their anti-predator defences in relation to ecological contexts. Therefore, increased efforts should be made to examine links between ecology, camouflage and behaviour and their influence on intraspecific colour divergence and local adaptation.

## Additional Information

**How to cite this article**: Marshall, K. L. A. *et al*. Microhabitat choice in island lizards enhances camouflage against avian predators. *Sci. Rep.* **6**, 19815; doi: 10.1038/srep19815 (2016).

## References

[b1] Hoekstra H. E., Hirschmann R. J., Bundey R. A., Insel P. A. & Crossland J. P. A single amino acid mutation contributes to adaptive beach mouse color pattern. Science 313, 101–104 (2006). 16825572 10.1126/science.1126121

[b2] McLean C. A., Moussalli A. & Stuart-Fox D. M. Local adaptation and divergence in colour signal conspicuousness between monomorphic and polymorphic lineages in a lizard. J. Evol. Biol. 27, 2654–2664 (2014). 25330209 10.1111/jeb.12521

[b3] Rosenblum E. B. Convergent evolution and divergent selection: Lizards at the White Sands ecotone. Am. Nat. 167, 1–15 (2006). 16475095 10.1086/498397

[b4] Rosenblum E. B., Hoekstra H. E. & Nachman M. W. Adaptive reptile color variation and the evolution of the MC1R gene. Evolution 58, 1794–1808 (2004). 15446431 10.1111/j.0014-3820.2004.tb00462.x

[b5] Gillis J. E. Substrate colour-matching cues in the cryptic grasshopper *Circotettix rabula rabula* (Rehn & Hebard). Animal Behaviour 30, 113–116 (1982).

[b6] Grant B. & Howlett R. J. Background selection by the peppered moth (*Biston betularia* Linn.): individual differences. Biol. J. Linn. Soc. 33, 217–232 (1988).

[b7] Kang C.-K., Moon J.-Y., Lee S.-I. & Jablonski P. G. Camouflage through an active choice of a resting spot and body orientation in moths. Journal of Evolutionary Biology 25, 1695–1702 (2012). 22775528 10.1111/j.1420-9101.2012.02557.x

[b8] Kang C.-K., Moon J.-Y., Lee S.-I. & Jablonski P. G. Moths on tree trunks seek out more cryptic positions when their current crypticity is low. Animal Behaviour 86, 587–594 (2013).

[b9] Kang C.-K., Stevens M., Moon J.-Y., Lee S.-I. & Jablonski P. G. Camouflage through behavior in moths: the role of background matching and disruptive coloration. Behavioral Ecology , doi: 10.1093/beheco/aru150 (2014).

[b10] Kettlewell H. B. D. Further background-choice experiments on cryptic Lepidoptera. Journal of Zoology 181, 371–376 (1977).

[b11] Kjernsmo K. & Merilaita S. Background choice as an anti-predator strategy: the roles of background matching and visual complexity in the habitat choice of the least killifish. Proceedings of the Royal Society, Series B 279, 4192–4198 (2012). 10.1098/rspb.2012.1547 PMC3441090 22915675

[b12] Nafus M. G. . Hiding in plain sight: a study on camouflage and habitat selection in a slow-moving desert herbivore. Behavioral Ecology , doi: 10.1093/beheco/arv096 (2015).

[b13] Sargent T. D. Background selections of geometrid and noctuid moths. Science 154, 1674–1675 (1966).

[b14] Grant B. & Howlett R. J. Background selection by the peppered moth (Biston betularia Linn.): individual differences. Biol J Linn Soc 33, 217–232 (1988).

[b15] Lovell P. G., Ruxton G. D., Langridge K. V. & Spencer K. A. Egg-laying substrate selection for optimal camouflage by quail. Current Biology 23, 260–264 (2013). 23333313 10.1016/j.cub.2012.12.031

[b16] Marshall K. L. A., Philpot K. E., Damas-Moreira I. & Stevens M. Intraspecific color variation among lizards in distinct island environments enhances local camouflage. PLoS ONE 10, e0135241 (2015). 26372454 10.1371/journal.pone.0135241 PMC4570707

[b17] Marshall K. L. A. & Stevens M. Wall lizards display conspicuous signals to conspecifics and reduce detection by avian predators. Behavioral Ecology 25, 1325–1337 (2014). 25419083 10.1093/beheco/aru126 PMC4235580

[b18] Marshall K. L. A., Philpot K. E. & Stevens M. Conspicuous male coloration impairs survival against avian predators in Aegean wall lizards, *Podarcis erhardii*. Ecology and Evolution 5, 4115–4131 (2015). 26442582 10.1002/ece3.1650 PMC4588654

[b19] Cooper W. E. & Sherbrooke W. C. Crypsis influences escape decisions in the round-tailed horned lizard (*Phrynosoma modestum*). Can J Zool 88, 1003–1010 (2010).

[b20] Cooper W. E. & Sherbrooke W. C. Choosing between a rock and a hard place: Camouflage in the round-tailed horned lizard *Phrynosoma modestum*. Current Zoology 58, 541–548 (2012).

[b21] Cuadrado M., Martín J. & López P. Camouflage and escape decisions in the common chameleon *Chamaeleo chamaeleon*. Biol J Linn Soc 72, 547–554 (2001).

[b22] Martín J., Luque-Larena J. J. & López P. When to run from an ambush predator: balancing crypsis benefits with costs of fleeing in lizards. Animal Behaviour 78, 1011–1018 (2009).

[b23] Rodríguez-Robles J. A., Leal M. & Losos J. B. Habitat selection by the Puerto Rican yellow-chinned anole, Anolis gundlachi. Can J Zool 83, 983–988 (2005).

[b24] Al-Hashem M. A. & Brain P. F. Changed substrate preferences shown by Fringe-toed Lizards, *Acanthodactylus scutellatus*, from Kuwait’s Al-Burgan oil field (Reptilia: Lacertidae). Zoology in the Middle East 46, 41–45 (2009).

[b25] Gibbons J. R. H. & Lillywhite H. B. Ecological segregation, color matching, and speciation in lizards of the *Amphibolurus decresii* species complex (Lacertilia: Agamidae). Ecology 62, 1573–1584 (1981).

[b26] Vetter R. S. & Brodie E. D. Background color selection and antipredator behavior of the flying gecko, Ptychozoon kuhli. Herpetologica 33, 464–467 (1977).

[b27] Stuart-Fox D., Whiting M. J. & Moussalli A. Camouflage and colour change: antipredator responses to bird and snake predators across multiple populations in a dwarf chameleon. Biol J Linn Soc 88, 437–446 (2006).

[b28] Vroonen J., Vervust B., Fulgione D., Maselli V. & Van Damme R. Physiological colour change in the Moorish gecko, *Tarentola mauritanica* (Squamata: Gekkonidae): effects of background, light, and temperature. Biol J Linn Soc 107, 182–191 (2012).

[b29] Martín J. & López P. When to come out from a refuge: risk-sensitive and state-dependent decisions in an alpine lizard. Behavioral Ecology 10, 487–492 (1998).

[b30] Martín J. & López P. Changes in the escape responses of the lizard *Acanthodactylus erythrurus* under persistant predatory attacks. Copeia 2003, 408–413 (2003).

[b31] Bauwens D., Hertz P. E. & Castilla A. M. Thermoregulation in a lacertid lizard: the relative contributions of distinct behavioral mechanisms. Ecology 77, 1818–1830 (1996).

[b32] Castilla A. M. & Bauwens D. Thermal biology, microhabitat selection, and conservation of the insular lizard *Podarcis hispanica astrata*. Oecologia 85, 366–374 (1990). 10.1007/BF00320612 28312041

[b33] Handrinos G. & Akriotis T. The Birds of Greece. (Christopher Helm Ltd., 1997).

[b34] Brock K. M., Bednekoff P. A., Pafilis P. & Foufopoulos J. Evolution of antipredator behavior in an island lizard species, *Podarcis erhardii* (Reptilia: Lacertidae): The sum of all fears? Evolution 69, 216–231 (2014). 25346210 10.1111/evo.12555

[b35] Runemark A., Brydegaard M. & Svensson E. I. Does relaxed predation drive phenotypic divergence among insular populations? Journal of Evolutionary Biology 27, 1676–1690 (2014). 24890841 10.1111/jeb.12421

[b36] Arnold N. & Ovenden D. Field Guide to Reptiles and Amphibians of Britain and Europe. (Harper Collins, 2002).

[b37] Grimm A., Ramírez A. M. P., Moulherat S., Reynaud J. & Henle K. Life-history trait database of European reptile species. Nature Conservation 9, 45–67 (2014).

[b38] Stevens M., Párraga C. A., Cuthill I. C., Partridge J. C. & Troscianko T. S. Using digital photography to study animal coloration. Biol J Linn Soc Lond 90, 211–237 (2007).

[b39] Troscianko J. & Stevens M. Image calibration and analysis toolbox – a free software suite for objectively measuring reflectance, colour and pattern. Methods in Ecology and Evolution , doi: 10.1111/2041-210X.12439 (2015). PMC4791150 27076902

[b40] Bergman T. J. & Beehner J. C. A simple method for measuring colour in wild animals: validation and use on chest patch colour in geladas (*Theropithecus gelada*). Biol J Linnean Soc 94, 231–240 (2008).

[b41] Stevens M., Stoddard M. C. & Higham J. P. Studying primate color: towards visual system-dependent methods. Int J Primatol 30, 893–917 (2009).

[b42] Verwaijen D. & Van Damme R. Wide home ranges for widely foraging lizards. Zoology 111, 37–47 (2008). 17997294 10.1016/j.zool.2007.04.001

[b43] Pike T. W. Using digital cameras to investigate animal colouration: estimating sensor sensitivity functions. Behav Ecol Sociobiol 65, 849–858 (2011).

[b44] Hart N. S. Vision in the peafol (Aves: *Pavo cristatus*). J Exp Biol 205, 3925–3935 (2002). 12432014 10.1242/jeb.205.24.3925

[b45] Hart N. S., Partridge J. C., Cuthill I. C. & Bennett A. T. D. Visual pigments, oil droplets, ocular media and cone photoreceptor distribution in two species of passerine bird: the blue tit (*Parus caeruleus* L.) and the blackbird (*Turdus merula* L.) Journal of Comparative Physiology 186, 375–387 (2000). 10798725 10.1007/s003590050437

[b46] Ödeen A. & Håstad O. The phylogenetic distribution of ultraviolet sensitivity in birds. BMC Evolutionary Biology 13, 36 (2013). 23394614 10.1186/1471-2148-13-36 PMC3637589

[b47] Castilla A. M., Gosá A., Galán P. & Pérez-Mellado V. Green tails in lizards of the Genus *Podarcis:* Do they influence the intensity of predation? Herpetologica 55, 530–537 (1999).

[b48] Castilla A. M. & Labra A. Predation and spatial distribution of the lizard *Podarcis hispanica atrata*: an experimental approach. Acta Oecologica 19, 107–114 (1997).

[b49] Sazima I. & D’Angelo G. B. The pale-breasted thrush (*Turdud leucomelas*) preys on a gekkonid lizard and an anomalepidid snake. Revista Brasileira de Ornitologia 19, 450–452 (2011).

[b50] Hart N. S. & Hunt D. M. Avian visual pigments: characteristics, spectral tuning, and evolution. Am Nat 169, S7–S26 (2007). 19426092 10.1086/510141

[b51] Vorobyev M. & Osorio D. Receptor noise as a determinant of colour thresholds. Proc R Soc B 265, 351–358 (1998). 10.1098/rspb.1998.0302 PMC1688899 9523436

[b52] Siddiqi A., Cronin T. W., Loew E. R., Vorobyev M. & Summers K. Interspecific and intraspecific views of color signals in the strawberry poison frog *Dendrobates pumilio*. J Exp Biol 207, 2471–2485 (2004). 15184519 10.1242/jeb.01047

[b53] Vorobyev M., Osorio D., Bennett A. T. D., Marshall N. J. & Cuthill I. C. Tetrachromacy, oil droplets and bird plumage colours. J Comp Physiol A 183, 621–633 (1998). 9839454 10.1007/s003590050286

[b54] Ruxton G. D. & Beauchamp G. Time for some a priori thinking about post hoc testing. Behav Ecol 19, 690–693 (2008).

[b55] Cabido C., Galán P., López P. & Martín J. Conspicuousness-dependent antipredatory behavior may counteract coloration differences in Iberian rock lizards. Behavioral Ecology 20, 362–370 (2009).

[b56] Stuart-Fox D. M., Moussalli A., Johnston G. R. & Owens I. P. F. Evolution of color variation in dragon lizards: Quantitative tests of the role of crypsis and local adaptation. Evolution 58, 1549–1559 (2004). 15341157 10.1111/j.0014-3820.2004.tb01735.x

[b57] Carter A. J., Goldizen A. W. & Tromp S. A. Agamas exhibit behavioral syndromes: bolder males bask and feed more but may suffer higher predation. Behavioral Ecology 21, 655–661 (2010).

[b58] Carter A. J., Heinsohn R., Goldizen A. W. & Biro P. A. Boldness, trappability and sampling bias in wild lizards. Animal Behaviour 83, 1051–1058 (2012).

[b59] López P., Hawlena D., Polo V., Amo L. & Martín J. Sources of individual shy–bold variations in antipredator behaviour of male Iberian rock lizards. Animal Behaviour 69, 1–9 (2005).

[b60] Stuart-Fox D. M., Moussalli A., Marshall N. J. & Owens I. P. F. Conspicuous males suffer higher predation risk: visual modelling and experimental evidence from lizards. Anim Behav 66, 541–550 (2003).

